# P-1372. Distribution and Mechanisms of Resistance among Carbapenem-Resistant *Enterobacterales* in Peru

**DOI:** 10.1093/ofid/ofae631.1549

**Published:** 2025-01-29

**Authors:** Yelinda V Reyes Quevedo, Aurora L Astocondor, Maribel D Riveros, Fiorella Krapp, Liliana Caytuiro Huamaní, Roxana Sandoval, Giancarlo Pérez-Lazo, Carolina Cucho, Iván Sabogal, Arnaldo Lachira, Kattya Michilot, Edwin A Cuaresma, Roberto R Diaz, Catherine Amaro, Alexander Briones, Araceli Olivares, Segundo A Burgos, Coralith Garcia, Manuel Melo, Stephanie Andrade, Luis A Hernández, Liz Lino

**Affiliations:** Instituto de Medicina Tropical Alexander von Humboltd-Universidad Peruana Cayetano Heredia, Lima, Lima, Peru; Instituto de Medicina Tropical Alexander von Humboldt, Universidad Peruana Cayetano Heredia, San Martín De Porres, Lima, Peru; Universidad Peruana Cayetano Heredia, PERU, Lima, Peru; Instituto de Medicina Tropical Alexander von Humboldt, Universidad Peruana Cayetano Heredia, San Martín De Porres, Lima, Peru; La Molina National Agrarian University, Lima, Lima, Peru; Hospital Nacional Guillermo Almenara Irigoyen, Lima, Lima, Peru; Hospital Nacional Guillermo Almenara Irigoyen, EsSalud, Lima, Lima, Peru; Dos de Mayo Hospital, Lima, Lima, Peru; Hospital Nacional Daniel A. Carrión, La Perla, Callao, Peru; Hospital III José Cayetano Heredia Red Asistencial EsSalud, Piura, Piura, Peru; Hospital III JOSÉ CAYETANO HEREDIA RED ASISTENCIAL DE ESSALUD PIURA, Piura, Piura, Peru; Hospital III Daniel Alcides Carrión Essalud Tacna, Tacna, Tacna, Peru; HOSPITAL REGIONAL LAMBAYEQUE, chiclayo, Lambayeque, Peru; Hospital Nacional Cayetano Heredia, Lima, Lima, Peru; Hospital regional de loreto, Iquitos, Loreto, Peru; Hospital Nacional Sergio E. Bernales, Lima, Lima, Peru; HOSPITAL REGIONAL DE PUCALLPA, PUCALLPA, Ucayali, Peru; Instituto de Medicina Tropical Alexander von Humboldt, Universidad Peruana Cayetano Heredia, San Martín De Porres, Lima, Peru; Hospital Nacional Dos de Mayo, Lima, Lima, Peru; Hospital Nacional Cayetano Heredia, Lima, Lima, Peru; Hospital Nacional Cayetano Heredia, Lima, Lima, Peru; Callao, Callao, Callao, Peru

## Abstract

**Background:**

Carbapenem-resistant *Enterobacterales* (CRE) are a public health threat due to their rapid worldwide spreading and the limited options to treat these infections.

Our aim was to describe the distribution and the type of carbapenemases produced by CRE isolates recovered in ten hospitals of Peru.

**Figure:**
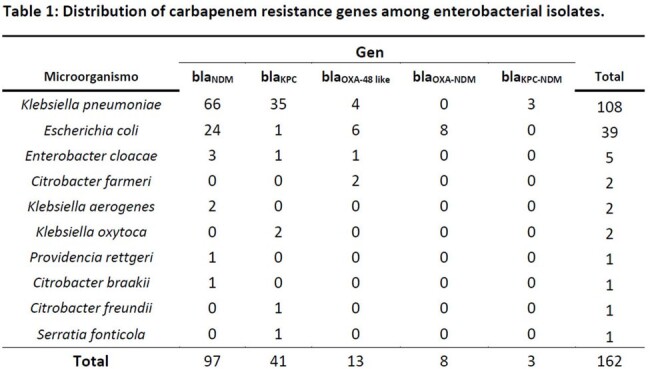

**Methods:**

A prospective collection of CRE isolates from clinical sources was obtained from 10 hospitals, located in Lima (capital of Peru) and other five Peruvian regions (Piura, Lambayeque, Loreto, Pucallpa and Tacna). Only one isolate per patient was included. The present work represents the first 171 consecutive isolates analyzed.

Identification and antimicrobial susceptibility were performed by the Phoenix ID-406 (Becton Dickinson, USA). For the determination of carbapenemase production the modified Carbapenem Inactivation Method was used. For the identification of carbapenemase types the NG-TEST CARBA-5, (Biotech, France) which identifies five carbapenemase families (*bla*_KPC_, *bla*_NDM_, *bla*_VIM_, *bla*_IMP_ y *bla*_OXA-48_), was used.

**Figure 1:**
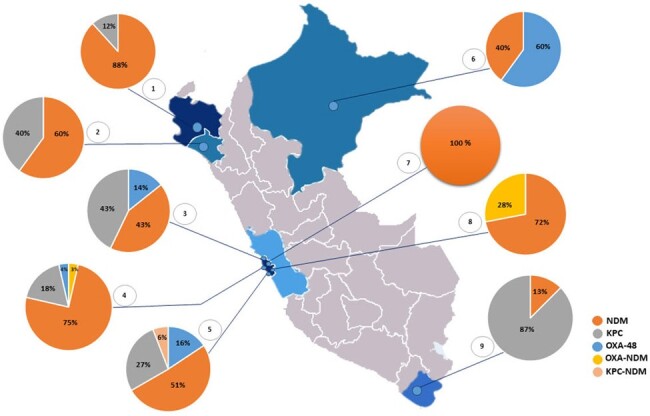
Distribution of Carbapenem resistance genes among hospitals surveilled. 1. Hospital III José Cayetano Heredia Red Asistencial EsSalud Piura; 2. Hospital Regional de Lambayeque, Lambayeque; 3. Hospital Nacional Cayetano Heredia, Lima; 4. Hospital Nacional Dos de Mayo, Lima; 5. Hospital Nacional Guillermo Almenara Irigoyen, Lima; 6. Hospital Regional de Loreto “Felipe Arriola Iglesias”, Loreto; 7. Hospital Nacional Sergio E. Bernales, Lima; 8. Hospital Nacional Daniel Alcides Carrión, Callao; 9. Hospital III Daniel Carrión de EsSalud, Tacna.

**Results:**

CRE isolates (n = 171) were recovered in 9/10 surveilled hospitals. Carbapenemase detection occurred in 94.7% of isolates distributed among 10 species, the most common ones were *Klebsiella pneumoniae* (66.6%) and *Escherichia coli* (24.0%) (**Table 1**).

NDM was the most common carbapenemase detected (59.9%) followed by KPC (25.3%) and OXA-48 (8.0%) distributed mainly among *K. pneumoniae* and *E. coli* isolates. Co-production of carbapenemases were identified in 6.8% of CRE isolates, including OXA+NDM among eight *E. coli* and KPC+NDM in three *K. pneumoniae* isolates (**Table 1**).

Regarding the distribution of carbapenemases, NDM was present in 9 hospitals and was the predominant in 6 of them; followed by KPC that was found in 6/9 hospitals and was predominant in one. OXA-48 was found in 4 hospitals (**Figure 1**).

**Conclusion:**

- The most common CRE in Peruvian hospitals are *K. pneumoniae* and *E. coli* species, accounting for 90% of all CRE isolates recovered from clinical sources.
- NDM is the most common carbapenemase, being predominant in 6 out of 9 surveilled hospitals.
- Double carbapenemase production was detected in over 6% of CRE isolates, both in *K. pneumoniae* and *E.coli* isolates.

**Disclosures:**

**Giancarlo Pérez-Lazo, n/a**, Pfizer: Grant/Research Support|Pfizer: Honoraria **Arnaldo Lachira, n/a**, MSD: Advisor/Consultant **Coralith Garcia, PhD**, Pfizer: Advisor/Consultant|Pfizer: Grant/Research Support

